# Feeding on Multiple Sources: Towards a Universal Parameterization of the Functional Response of a Generalist Predator Allowing for Switching

**DOI:** 10.1371/journal.pone.0074586

**Published:** 2013-09-25

**Authors:** Andrew Morozov, Sergei Petrovskii

**Affiliations:** Department of Mathematics, University of Leicester, Leicester, United Kingdom; University of Florida, United States of America

## Abstract

Understanding of complex trophic interactions in ecosystems requires correct descriptions of the rate at which predators consume a variety of different prey species. Field and laboratory data on multispecies communities are rarely sufficient and usually cannot provide an unambiguous test for the theory. As a result, the conventional way of constructing a multi-prey functional response is speculative, and often based on assumptions that are difficult to verify. Predator responses allowing for prey selectivity and active switching are thought to be more biologically relevant compared to the standard proportion-based consumption. However, here we argue that the functional responses with switching may not be applicable to communities with a broad spectrum of resource types. We formulate a set of general rules that a biologically sound parameterization of a predator functional response should satisfy, and show that all existing formulations for the multispecies response with prey selectivity and switching fail to do so. Finally, we propose a universal framework for parameterization of a multi-prey functional response by combining patterns of food selectivity and proportion-based feeding.

## Introduction

Population dynamics of multi-species communities is a major challenge in contemporary ecology [1]–[5]. In particular, parameterization of the functional response of a generalist predator feeding on multiple resources has been a focus of ecological literature for a few decades already and various approaches have been suggested [6]–[12]. It has previously been shown that different forms of the response can result in different predictions for the dynamics of the community [8], [13], [14]; therefore, an understanding of this issue is central to community and food web ecology. However, while the dynamics of few-species systems (*n
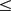
*3) have by now been studied almost comprehensively (but see [15], [16]), good understanding of multi-species systems (that can include hundreds or even thousands of species) is still lacking.

Parameterization of a predator functional response for an arbitrary number of food sources can be derived using various theoretical approaches. For instance, it can utilize the optimal foraging theory framework [8], [10], [17]–[22]; it can be based on specific biological traits such as the predator's memory [23] or simply use common-sense reasoning [11], [12], [14], [24]–[26]. Empirical verification of different parameterizations, however, remains a considerable challenge: although experimental data on feeding of predator/consumers on multiple resources are abundant both in marine and terrestrial ecology [27]–[34], laboratory experiments and field observations are mostly limited to the case where the predator has the choice of only a few varieties of food (*n
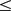
*4). On the other hand, ecosystems often contain a large number of species belonging to the same trophic level. Moreover, even within a single prey population individuals can largely differ in terms of size/age and behavior (e.g. [35]) so that, from the predator's point of view, they are likely to be different food sources. There can be also a pronounced variation in terms of the parasite load that organisms carry and predation can heavily depend on the degree of infectivity of prey [36]. Thus, in real ecosystems there can be hundreds or thousands of food sources which are, from the predator's perspective, either similar or distinctly different.

Construction of a multi-prey functional response in the presence of a large variety of resources is not straightforward, as it always requires some hypotheses about the predator foraging behavior that can be species-specific and are very difficult or even impossible to test experimentally (cf. [11]). This uncertainty has resulted in a broad variety of different mathematical formulations of functional response, each them having some rationale behind the equations; see [8] and [11] for a review. The type of response which is regarded as simplest is the so-called proportion-based consumption where individual predators pick up the prey items randomly and thus the food intake rate is determined by the relative frequency of the prey species in the community [11], [37], [38]. However, the proportion-based response is often thought to miss the complexity of foraging behavior [6], [8]. Eventually, more sophisticated parameterizations were developed to describe food intake by animals with complex foraging strategies, e.g. exhibiting food selectivity and active switching. In this case, a predator can ‘switch’ towards a more energetically profitable food source, ignoring perhaps more abundant but less beneficial prey items [21], [27], [29], [33], [39]–[41]. A widespread opinion is that predator responses allowing for switching are more relevant than proportion-based ones because they enhance the stability of food webs and promote biodiversity [9], [14], [18], [42], [43].

In spite of considerable work done on food selectivity and switching (see the references above), several new parameterizations of the predator functional response have recently been suggested [12]–[14], [23], [43] as part of the attempt to explain the patterns of biodiversity in complex communities, in terms of top-down control by predators, such as zooplankton in plankton ecosystems [44], [45]. This situation is somewhat worrying as it considerably increases the uncertainty in the choice of the theoretical/modeling framework. It seems obvious that one can construct infinitely many different mathematical functions or their combinations to describe a predator response, each of them having some rationale, however, unless a set of biologically sound criteria is developed, it is not immediately clear which parameterization is better than the others and why. The main goals of this paper are to perform a critical analysis of the predator functional responses found in the literature and to formulate a set of general, basic criteria or rules which a predator functional response should satisfy, whether it includes switching or not. We apply these basic rules to the existing functional responses and show that, whereas most of the proportion-based functional response formulations satisfy these rules, the admittedly ‘more advanced’ functional responses with food selectivity and switching often fail to do so. Furthermore, we demonstrate that the existing formulations of the functional response may result in unrealistic predictions, in particular when they are used to relate the biodiversity and ecosystems' productivity.

A point that we want to make in this paper is that, in spite of their apparent biological relevance, functional responses with food selectivity has their own limitations. Predator switching between two or more prey species is only possible when the prey species are, from the perception of the predator, sufficiently different. Indeed, if the predator cannot detect any difference between different types of prey, there is no reason for switching; therefore, predator feeding on similar prey species has to follow the proportion-based consumption. In the following, we refer to the prey species that may be undistinguishable from the predator's point of view as species with similar life traits. Ultimately, one may consider a hypothetical situation where individuals of the same prey species are marked with different markers in order to split the prey population into several groups: in the case where the markers are unperceivable to the predator, its choice of prey items is going to be perfectly random and hence has to follow the proportionate rule.

Correspondingly, we argue that using a predator response with switching in communities with a large number of prey species can be conceptually wrong. Consider a plankton system as a paradigm of a multi-species community. Plankton communities often consist of hundreds, even thousands of species [46]–[50], with many of them having similar life traits. This means that the spectrum of food resources that a given zooplankton species feeds on is likely to include very different prey species as well as similar species. An appropriate parameterization of the zooplankton (predator) response should be then of a transitional type, i.e. allowing for selectivity and switching when feeding on significantly different species, but turning to a proportionate response when feeding occurs on similar species. The same holds true for modeling trophic interaction in some other systems with high biodiversity such as insect communities [51] or coral reef ecosystems [52].

The paper is structured as follows. In Section 2, we introduce a set of rules that the multi-prey functional response of a predator should satisfy. In Section 3, we carefully examine the existing formulations of a multi-prey functional response and show that none of them satisfies these rules. In Section 4, we show how the drawbacks of the previous formulations of functional responses can be amended, resulting in a transitional parameterization of the predator's intake rate which describes consumption of prey with both close and distinctly different life history traits. In Section 4, we discuss the implications of our findings; in particular, we show how are results may contribute to the recent efforts in the literature to relate the biodiversity and productivity of ecosystems.

## Results

### Basic rules for a multi-prey functional response

Throughout this paper, we assume that the predator functional response is prey-dependent. A known alternative is the ratio-dependent response that takes into account interference between predators [53], [54]. However, inter-predator interference is likely to become important only when the predator population density is very high. Considering the plankton community as a paradigm of a multispecies system, phytoplankton (i.e. prey) outbreaks are well known and relatively frequent phenomena, cf. “red tides” and “green tides” as well as seasonal phytoplankton blooming [47]. On the contrary, mesozooplankton (predator) outbreaks up to the densities where the presence of other foragers could strongly affect individual intake rate are very rare (e.g. [55]). We therefore restrict our analysis to the cases when the predator density is not very high without any substantial loss of generality.

We consider that the single resource functional response (i.e. in the absence of all other resources) is described by the function *f_0i_*(*P_i_*), where *P_i_* is the density of resource *i*. Function *f_0i_*(*P_i_*) therefore describes the per capita rate of increase of a given ‘consumer’ species when it feeds on the resource *i* only. This rate of increase can be affected by the presence of other resources. Correspondingly, we denote the multiple resource functional response (describing the intake rate of resource *i* in the presence of other resources) by 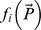, where 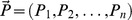.

In the below, we will refer to the consumer species as predator and to the resource species as prey. We shall introduce several criteria or rules that are meant to account for biologically reasonable properties of the predator functional response and to avoid artificial or unrealistic behavior. In order to reveal any potential artifacts, we apply a special test: we consider a thought experiment where the given prey species is split into several groups or subpopulations in such a way that an observer can distinguish between the groups but the predator cannot. In reality, such splitting could be done by using certain chemical markers or radioactive tracers. The idea of this thought experiment is that, since all those groups consist of essentially the same prey species, a ‘good’ functional response should be stable with respect to such splitting while a ‘bad’ one would not.

We begin with the rule that is known in the ecological literature as the consistency requirement condition [23], [56]. Here we use it in the following form:

#### Rule (i)

*A biologically reasonable parameterization for the multi-prey functional response 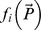 should be applicable to the case where all the resources have similar properties.*

Here “similar properties” means that, ultimately, different resources may be indistinguishable from each other. Following the idea of our thought experiment, let us consider that a single prey species is divided arbitrarily into *N* groups. Obviously, each of these groups has exactly the same parameters and hence their consumption by the predator ought to be described by the same function. In this case, the following identity should apply:



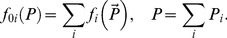
(1)


In other words, for a sound functional response splitting the same species into several groups and summing back the consumption rates should give the same consumption rate as for the single species with the overall density *P*. Note that it does not matter whether the prey species in different groups is actually the same or only “similar” as long as the predator cannot spot the difference. Therefore, Rule (i) should apply as well in the situation, where the prey species are morphologically different but from the predator perception they are similar as potential food resources.

Rule (i) addresses the case of feeding on a single prey species or on several prey species with similar traits. Generally speaking, the presence of alternative resources with distinctly different traits can change the situation. However, similarly to the above, the predator's food consumption of a given resource (prey) should still be stable with respect to its division. Hence we arrive at the following rule:

#### Rule (ii)

*Parameterization 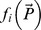 should remain valid when resource i is split into m groups in the presence of other resources.*

Rule (ii) therefore gives an extension of Rule (i). As we will show below, this extension is nontrivial and goes beyond the basic consistency requirement condition. Splitting the *i*th resource into *m* groups can reflect some actual intraspecific variation of certain life traits but it can just as well be a part of the thought experiment. In order to obtain a mathematical formulation of Rule (ii), we denote the resource partition in the system before splitting as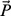, and after splitting as 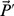, with 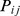 being the density of each group of resource *i*. The total intake rate of resource *i* (now consisting of *m* groups) should then satisfy the following equation:
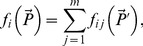
(2)


or, equivalently,
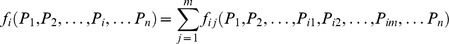



Therefore, splitting the *i*th prey species into *m* groups – now in the presence of alternative prey species – and summing back the consumption rates over the groups should give the original consumption rate for the resource *P_i_*. We want to emphasize that satisfaction of the logical consistency requirement as given by Rule (i) does not, in general, guarantee satisfaction of Rule (ii). In Section 3 we will show an example of such situation.

#### Rule (iii)

*Predation on any two species with close life traits cannot change the ratio of their population densities, which will therefore remain constant over time.*

Indeed, consider the situation when a predatory species feeds on two prey species with close life traits. For the sake of simplicity we assume that there is only one predator. (Following the same approach as above, ultimately, we can consider the population of the same prey species with population density *P* split into two groups with densities *P_1_* and *P_2_* respectively.) In this case, the predator cannot distinguish between the groups and hence it is bound to proportionate consumption. Correspondingly, the initial ratio of *P_1_* and *P_2_* should remain the same over time, i.e. *P_1_*/*P_2_*≈*const*, which we can write as
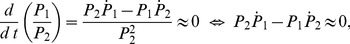
(3)


The temporal dynamics of each of these species can be described by the following equation which is a standard equation in food web models (e.g. [57] also see Eqs.6–8 below):
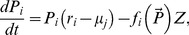
(4)where *r_i_* and *µ_i_* (*i* = 1,2) are the growth and mortality rates of prey and *Z* is the density of predator. Equation (4) accounts for the fact that the change in the prey density is due to growth, mortality and predation. Since the two prey species are assumed to have similar traits, their per capita growth rates and mortalities have close values, i.e. *r*_1_≈*r*_2_ and *μ*_1_≈*μ*_2_. Using (4), we then can re-write condition (3) as



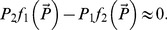
(5)
Equation (5) thus gives a mathematical expression of Rule (iii).

In a more general case of *N* species with close traits (or by splitting the whole population into *N* parts), we can easily derive a condition analogous to (5) for any pair of *P_i_* and *P_j_*, 1≤ *i*, *j*≤*N*. Expression (5) should therefore hold for all prey species that are different but have close life traits; in particular, when *r*_i_≈*r*_j_ and *μ*_i_≈*μ*_j_.

#### Rule (iv)

*The total predicted biomass of prey and/or predator in generic food chain models should not be sensitive to the way in which the species are divided into groups*.

Let us consider a generic food web consisting of a large number of competing prey species. A standard model is given by the following equations (cf. [12], [57], [58]):
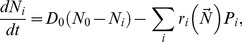
(6)

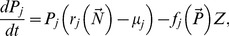
(7)

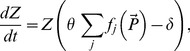
(8)where *N_i_* is the density of the limiting nutrients; *P_i_* are the densities of prey (e.g. phytoplankton), *i* = 1, …, *n* (*n*>>1); Z is the density of predator (e.g. zooplankton). For the sake of simplicity we consider that there is only one predator. Parameter *θ* is the food utilization coefficient, *δ* is the mortality of the predator, *r_i_* and *μ*_i_ are the growth rate and mortality of the prey species *i*, respectively, and *D*_0_ characterizes the rate of replenishment rate of nutrients (e.g. due to vertical diffusion in the water column).

A common sense expectation is that that the stationary total amount of prey *P* and predator *Z* (determining ecosystem productivity) as obtained from (6)–(8) or any similar generic food chain model should not be sensitive with respect to the way we subdivide the prey species into subpopulations or groups. Indeed, such division can be purely conventional or even arbitrary (cf. the thought experiment above) and hence it should not determine the essential system properties. In particular, if we increase the number *n* by splitting each prey species *i* in to *m_i_* groups with the same life traits, this should not affect the total biomass of the given trophic level. Violation of Rule (iv) can result in an artificial bias in the relation between productivity and biodiversity which is predicted by some models; see examples in Section 5.

#### Rule (v)

*The total food intake rate should be an increasing function of densities of each of the resources. Mathematically this condition can be expressed as *

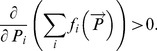
(9)


Functional responses satisfying this condition are known in the literature as optimal functional responses. A functional response which does not satisfy Rule (v) is called suboptimal. Only an optimal functional response can have a solid ecological and evolutionary rationale [8], [11], [59]. Indeed, a suboptimal response effectively assumes that an extra food may act as a poison. A drop in the overall intake rate when a resource becomes more abundant can hardly have a solid theoretical justification and should be considered as a mathematical artifact [11], [59].

#### Rule (vi)

*Use of Holling type III to describe pairwise predator-prey interactions should be avoided unless it is carefully documented and justified.*

For a given predator species, one should verify which type of response (according to the well-known Holling classification) takes place for feeding on a single food source. In particular, it has been argued [6], [39], [41] that a sigmoid functional response known as Holling type III may be a fingerprint of active switching: the intake of a given resource by the predator can drop quickly at small resource densities as a result of the predator switching to an alternative prey species, even if the latter is not considered explicitly. However, in the case where we explicitly describe *all* the possible resources in the system, the use of a sigmoid parameterization may not always be justified. A potential problem may occur in the case where the densities of all resources attain low values: since there are not any alternative resources, a simultaneous sharp drop in all 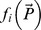 would then be a model artefact. Therefore, unless there is a clear argument supporting the use of Holling type III, Holling type II should be used.

### Critical analysis of the existing formulations of the multi-prey functional response with switching

As a part of our critical analysis we have considered several multi-prey functional responses for a predator with food selectivity and switching which we found in the literature [8], [11]–[14], [23]–[26], [34], [60]–[65]. We have checked whether Rules (i)–(vi) are satisfied. Our results are summarized in Table 1 and details can be found in the online appendix; see Material S1. Rather surprisingly, none of the existing multi-prey functional responses with switching behavior satisfies all basic Rules (i)–(vi) indicating that those parameterizations may have some hidden caveats. In particular, most parameterizations (except for the one by [23] which we will analyse separately, see below) fail to account for the situation where species possess close life traits. Splitting a single population into groups and summing them back gives the initial functional response only in the absence of other resources; in particular, it does not work in the case of two distinctly different prey species.

**Table 1:** Results of testing the existing multi-prey predator's functional responses against the set of the basic Rules (i)–(vi). For details, see the text and the online appendix in Material S1.

Functional response, reference	Parameterization	Rule (i)	Rule (ii)	Rule (iii)	Rule (iv)	Rule (v)	**Rule (vi) (Holling type for a single resource)**
[12], [14] and [64]	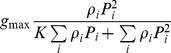	NO	NO	NO	NO	NO	Type II
[26]	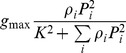	NO	NO	NO	NO	YES	Type III (sigmoid)
[24]	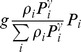 _(*γ*>1)_	NO	NO	NO	NO	NO	Type I (can be extended to type II)
[62]	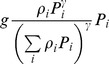 _(*γ*>1)_	NO	NO	NO	NO	NO	Type I (can be extended to type II)
[65] (generalized formulation)	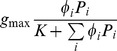 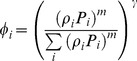	NO	NO	NO	NO	NO	Type II
[63]	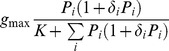	NO	NO	NO	NO	YES	Type II for *Kδ*<1, otherwise type III
This paper, see also [71]	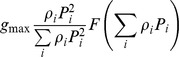	YES	NO	NO	NO	YES	Types I, II, III (Depending on *F*)
[23]	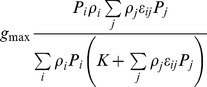	YES	YES	YES	YES	Supp. onstrains required on *ε_ij_*	Type II
This paper, Eqs.(12), (15–16)	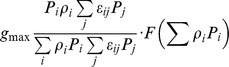	YES	YES	YES	YES	YES	Types I, II, III (Depending on *F*)
[11]	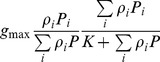	YES	YES	YES	Not applicable	YES	Type II

Interestingly, the functional responses with proportion-based consumption ([11] see Table 1) satisfy all the basic rules (except Rule (iv) since in this case we do not have the possibility of coexistence of more than two species due to competitive exclusion) and hence are able to correctly describe consumption of prey species with similar traits. However, they fail to take into account switching.

Rule (**vi**) (Holling type for a single resource) is not satisfied for the parameterization suggested by [26]. Their parameterization is essentially of Holling type III and describes feeding of herbivorous zooplankton; however, this seems to be at odds with many field and laboratory studies. Indeed, a large number of laboratory experiments prove non-sigmoid Holling types I or II functional responses for zooplankton grazers [38], [66]–[68]. This is particularly true for the most efficient zooplankton grazers – microzooplankton- which usually exhibit non-sigmoidal Holling types I and II responses [69], [70]. Assuming a sigmoid response for zooplankton grazers can therefore result in a substantial underestimation of the intake rate at low overall biomasses of phytoplankton in models.

Recently, a new parameterization of the functional response was proposed in [23]. It was derived based on the biologically reasonable assumption that the predator possesses memory of the quality of the prey consumed previously, which defines its choice of the next prey item. Correspondingly, their parameterization seems to be more advanced compared to the previous attempts; indeed, as it is shown in Table 1, it satisfies Rules (i–iv) and (vi). However, optimality remains a problem: it is readily seen (see Material S1 for details) that Rule (v) does not hold unless some special constraints on the food similarity parameters *ε_ij_* are introduced. In particular, for two distinctly different species (say, species 1 and species 2), the optimal intake is only possible for *ε_12_*> max (*ε_22_*, *ε_11_*)/2. The applicability of the parameterization given in [23] is therefore restricted to communities with special properties.

Moreover, a biologically meaningful functional response with food selectivity should account for the food selectivity, which is a function of relative proportions of food densities [11] Mathematically, in case of a very low or a very high relative proportion of resources, it means that


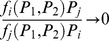
 for 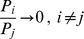 (11)

It is readily seen (see the online appendix for details) that, for the functional response proposed in [23], we obtain that, for *P_i_*/*P_j_* →0, *f_i_P_j_*/(*f_j_ P_i_*) → *ε*_12_/*ε*_jj_. Apparently, it can only be consistent with food selectivity or switching if *ε*_12_<<*ε*_jj_; see Eq.(11). However, this clearly disagrees with the condition on the food similarity coefficients needed for optimality, see the previous paragraph, as the latter implies that *ε*_12_/*ε*_jj_>1/2. Therefore, for the functional response in [23], optimality and food selectivity appear to be mutually exclusive, at least in the range of large or small values of the prey population density. As a result, under the optimality condition, their parameterization of the functional response describes a proportionate resource consumption with no selectivity, except for relatively close values of *P_1_* and *P_2_*.

The fact that there is no switching for small and large ratios of resource densities can have a major effect on model predictions. In particular, the per capita mortality due to predation on a species with very small density *P_i_* will remain non-zero as in the classical proportion-based consumption [11], [14] whereas in the case of (11) the rare species will be released from predation. At the same time, we want to mention it here that the fact that a functional response does not satisfy condition (11) does not necessarily mean that the corresponding parameterization is totally irrelevant or unrealistic. However, its switching properties do become restricted, especially in the important case where the density of one resource is much larger or smaller compared to the others. We therefore conclude that the problem of finding an appropriate parameterization for the predator multispecies response remains open. We address it in the next section.

### Transitional multi-species functional response combining switching with proportion-based feeding

We shall now make an attempt to derive a universal, transitional functional response combining the features of both active and passive feeding. In doing this, we also aim to demonstrate that it is possible to construct a multispecies response that satisfies all Rules (i–vi), i.e. the constraints given by the rules are not mutually exclusive. As a starting point, we use the generic family of multi-prey responses as given in [11], [13], which has the following form:
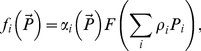
(12)where *α_i_* have the meaning of food preferences (which can be either constant or certain functions of 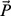), Σ *α_i_*  = 1, and *F* is a function which describes the total consumption of all resources (e.g. Holling type II);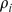 are positive parameters (having the meaning of weights, however the sum of 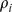 can be different from 1).

Depending on the choice of *α_i_*, response (12) can attain different properties. In particular, it describes the proportion-based consumption if we choose
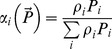
(13)


(cf. [11]), and it can allow for switching if we choose *α_i_* differently. For instance, it is readily seen that the functional response with switching used in [71] (which is essentially a generalization of the previous results by [11] and [24]) coincides with (12) if we choose
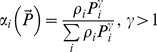
(14)Here we suggest the following parameterization of *α_i_*

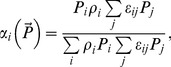
(15)where ε_ij_ are coefficients describing similarities between different types of food.

As we have argued above, for similar resources, i.e. for prey species with close life traits, consumption should be proportion-based (13), whereas for substantially distinct food sources the preference should be described by selectivity. In order to quantify closeness of different food sources in terms of the ability of the predator to distinguish between those sources, we introduce a certain parameter ω. This parameter describes a relevant species trait, for instance, the body size of prey, the defensive ability of prey, the body shape, mobility of organism, etc. (In a more general case ω can be a vector taking into account many different traits.) Thus, similar types of food sources *i* and *j* should have close values *ω_i_* and *ω_j_* whereas distinct resources should have substantially different values.

Our main hypothesis here is that the food similarity coefficients are functions of their closeness so that for close prey species *ε_ij_* >>0 and *ε_ij_* ≈0 for distinctly different prey species. For the sake of simplicity and to lessen the number of parameters, we consider that *ω_i_ = ρ_i_*, thus assuming that similarity between food sources is sufficiently taken into account by the weights *ρ_i_*. For the functions *ε_ij_* one can use, for instance, the Gaussian distribution.
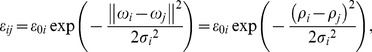
(16)where *ε_0i_* is the normalizing coefficient (because the sum of *ε_ij_* over *j* should be equal to unity) and *σ* is a parameter which depends on the sensitivity of the predator.

Now we demonstrate how the transitional parameterization (12), (15) and (16) works for the two important limiting cases.

*Case A: all prey species are similar*. Different prey species are not distinguished by the predator. In this case for all prey species we have *ρ_i_≈ρ* = *const* and *ε_ij_≈ ε = const*. From (12), (15–16), after some standard calculations we obtain:
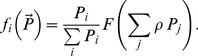
(17)


Equation (17) means that close species are consumed according to the proportion-based relation, which is exactly what should intuitively be expected. Note that even in the presence of other, distinctly different resources (i.e. in the case when not all *ρ* are equal) those prey species that have the same *ρ* will be consumed according to the proportion-based law. In other words, the relative ratio of consumption rates will be given by
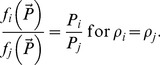
(18)


*Case B: distinctly different species.* For a distinct set of resources quantified by *ρ*_1_, *ρ*_2_, …, *ρ*_n_, respectively, *σ_i_* can be considered as very small compared to the distances between the species, i.e. *σ_i_* << *ρ_i_*. In this case, the kernel (16) approaches the Dirac delta function centered at *ρ*_i_ and summation 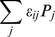 give *P_i_*, so that (12), (15–16) turns into


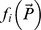
  =  
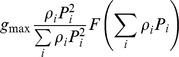
which clearly describes a response with switching. Thus, we have prey switching by the predator in the case of feeding on distinctly different resources.

Finally, it is easy to check that the functional response (12), (15–16) satisfies all the basic rules introduced in Section 2. Rules (i) and (ii) are satisfied by construction of 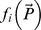; Rule (iii) is satisfied because for close species the consumption is described by the proportion-based formulation (13), for which this rule holds. The other rules are also satisfied, in particular, the a universal, transitional functional response is suggested optimal (in the sense of Rule (v)) since the overall summation of (12) will give the resultant function *F* which we assume to be an increasing function of the food density.

## Discussion

The choice of parameterization for the predator functional response is a key issue for modelling food web dynamics. It has attracted considerable attention recently, in particular because of the attempts to relate patterns of biodiversity in complex communities with ecosystem productivity/biomass [12], [44], [45], [72]. In a recent simulation study, Prowe et al. (see [12], [14]) showed the existence of a strong positive correlation between the species richness and the biomass of primary producers in ecosystems with top-up control. In [14] it was found that the total biomass of primary producers 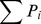 in complex multi-species planktonic ecosystems exhibits a rapid increase in response to an increase in the species richness *n*. This was observed both in a non-spatial and 3-D spatial models (see fig 4 and fig 8 in the cited paper). For example, transition from 4 to 20 primary producers would result in an increase in the total biomass approximately by a factor of 3.5. Moreover, a severe drop in the nutrient level was reported as a consequence of an increase in the system richness. However, a close inspection of the predator functional response used in [14] shows that it is not stable with respect to species sub-division; in particular, Rule (iv) above is not satisfied. A several-fold time increase in the total biomass could be observed if a single phytoplankton species is split into several groups that are indistinguishable to consumers (see the description of the thought experiment in Section 2). Our analysis based on the basic food chain model (6)–(8) (see Material S1) can easily explain the findings in [14]. Indeed, one can see that 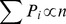 within a certain range of *n* (see (S9) and (S9′) in Material S1). Analysis of the expression for the level of the limiting nutrient *N* shows (see (S8) in Material S1) that it is a decreasing function of species richness *n*. Correspondingly, one can observe a similar increase in biodiversity and transmission to oligotophic conditions (a severe drop in *N*) not by increasing the species richness but by just splitting a single phytoplankton species into *n* groups. It indicates that the findings [14] can be specific to their choice of the functional response, effectively being a model artefact.

Therefore, one should be very careful when choosing a parameterization of the functional response of a generalist predator in multi-species models as it may affect the model predictions. We should mention here that, even in the simple case where the predator consumes only one type of resource, variation in the mathematical formulation of the functional response can affect the model properties significantly [73]–[75], a phenomenon known as *structural sensitivity* of biological models [75], [76]. Considering predation on multiple resources makes food web models even more sensitive to the choice of functional response parameterization [13], [8], [64]). However, unlike the case of a single-prey functional response, the conventional way to choose the parameterization of a multi-prey functional response is mostly theoretical [11], [59], mainly due to limited empirical data.

In this paper, we endeavour to make the process of selection of the functional response more rational by introducing certain basic rules (see Section 2) specifically designed in order to avoid model artefacts. In particular, we argue that, in truly multi-species systems (such as plankton communities [46]; [48]), insect communities [51] or coral reef ecosystems [52], one can hardly reduce the biological rationale behind the functional response to the case of clearly different resources allowing for food selectivity of the predator. In the cases where a consumer has to choose between hundreds or even thousands of different food sources, the predator will often be unable to distinguish between prey species with close life traits. Hence it will show switching only between distinctly different food sources but has to employ proportion-based feeding on close food sources. Surprisingly, this argument seems to have been somewhat ignored in the derivation of the most of the recently proposed functional responses (but see [23]).

We propose a novel theoretical framework for construction of a transitional multi-prey functional response (12), (15) and (16) which combines food selectivity and switching with proportional-based consumption. We quantify the entire spectrum of different resources according to their relative closeness to each other, i.e. food resources with close types are (virtually) undistinguishable by the predator. Introducing similarity of the resources has an advantage over the previous formulations as it allows us to combine switching for distinct types of food with proportion-based consumption for similar types into a single mathematical expression. The new multi-prey functional response (12), (15) and (16) satisfies Rules (i)–(vi). Note that our approach can be readily extended to the case of a continuous spectrum of resources (as can arise, for instance, when one takes into account an inherently continuous statistical variation of individual traits, cf. [77]).

We mention here that the rules introduced in this paper (see Section 2) are necessary but by no means exhaustive. Particularities of a given ecosystem and of specific prey and/or predator species may impose supplementary requirements and constraints on the choice of the functional response parameterization. For instance, any interference between the predators (e.g. [54], [64]) is likely to affect their prey selectivity. Also, the existing parameterizations of the predator functional response mostly focus on the resource densities; however, the handling times are likely to have a similar effect on the predator's choice of food. Indeed, an optimal strategy for a predator would likely be to consume the resources with a small handling time and to avoid those with large handling times [78], especially if the predator has a sufficiently long memory (cf. [23]) and the resources are abundant, so food handling would basically determine the entire intake rate. It seems reasonable to expect that the predator then should exhibit switching, at least when it feeds on a few resources that are sufficiently different in terms of their handling time. Surprisingly, it is readily seen that none of the functional responses listed in Table 1 is capable to describe switching with respect to the handling time, all of them showing only proportion-based prey consumption. A better understanding of this issue should become a focus of future research.

## Supporting Information

Material S1 Verification of the Basic Rules for the multi-prey functional responses listed in Table 1.
(DOC)

## References

[1] Steinke M, Malin G, Liss PS (2002) Trophic interactions in the sea: An ecological role for climate relevant volatiles? J Phycol 38: 630–638.

[2] Cohen JE, Jonsson T, Carpenter SR (2003) Ecological community description using the food web, species abundance, and body size. Proc Natl Acad Sci USA 100: 1781–1786. 12547915 10.1073/pnas.232715699 PMC149910

[3] Odum EP, Barrett GW (2005) Fundamentals of ecology. Thompson Brooks/Cole, Belmont, CA.

[4] Begon M, Townsend CR, Harper JL (2005) Ecology: from individuals to ecosystems, 4th Edition. Michael. 752p.

[5] Massol F, Gravel D, Mouquet N, Cadotte MW, Fukami T, et al (2011) Linking community and ecosystem dynamics through spatial ecology. Ecol Lett 14: 313–323. 21272182 10.1111/j.1461-0248.2011.01588.x

[6] Murdoch WW, Oaten A (1975) Predation and population stability. Adv Ecol Res 9: 1–131.

[7] Comins HN, Hassell MP (1976) Predation in multi-prey communities. J Theor Biol 62: 93–114. 994522 10.1016/0022-5193(76)90053-9

[8] Holt RD (1983) Optimal foraging and the form of the predator isocline. Amer Nat 122: 521–541.

[9] Armstrong RA (1999) Stable model structures for representing biogeochemical diversity and size spectra for plankton communities. J Plankton Res 21: 445–64.

[10] Krivan V, Sikder A (1999) Optimal foraging and predator-prey dynamics II. Theor Popul Biol 5: 111–126. 10.1006/tpbi.1998.1399 10329511

[11] Gentleman W, Leising A, Frost B, Storm S, Murray J (2003) Functional responses for zooplankton feeding on multiple resources: a review of assumptions and biological dynamics. Deep-Sea Res II Top Stud Oceanog 50: 2847–2875.

[12] Prowe AEF, Pahlow M, Dutkiewicz S, Follows M, Oschlies A (2012a) Top-down control of marine phytoplankton diversity in a global ecosystem model. Prog Oceanogr 101: 1–13.

[13] Anderson TR, Gentleman WC, Sinha B (2010) Influence of grazing formulations on the emergent properties of a complex ecosystem model in a global Ocean general circulation model. Prog Oceanogr 87: 201–213.

[14] Prowe AEF, Pahlow M, Oschlies A (2012b) Controls on the diversity productivity relationship in a marine ecosystem model. Ecol Model 225: 167–176.

[15] Erbach A, Lutscher F, Seo G (2013) Bistability and limit cycles in generalist predator-prey dynamics. Ecol Complex 14: 48–55.

[16] Hilker FM, Liz E (2013) Harvesting, census timing and “hidden” hydra effects. Ecol Complex 14: 95–107.

[17] McNair JN (1981) A stochastic foraging model with predator traning effects. II Optimal diets. Theor Pop Biol 19: 147–162. 10.1016/0040-5809(80)90003-9 7404444

[18] Abrams PA (1982) Functional responses of optimal foragers. Amer Nat 120: 382–390.

[19] Hutson V (1984) Predator mediated coexistence with a switching predator. Math Biosci 68: 233–246.

[20] Stephens DW, Krebs JR (1986). Foraging theory. Princeton University Press, Princeton, New Jersey, USA.

[21] Krivan V (1996) Optimal foraging and predator-prey dynamics. Theor Popul Biol 49: 265–290. 8813025 10.1006/tpbi.1996.0014

[22] Baalen M, Křivan V, van Rijn PCJ, Sabelis MW (2001) Alternative food, switching predators, and the persistence of predator-prey systems. Amer Nat 157: 512–524. 18707259 10.1086/319933

[23] Van Leeuwen E, Brännström A, Jansen VA, Dieckmann U, Rossberg AG (2013) A generalized functional response for predators that switch between multiple prey species. J Theor Biol 328: 89–98. 23422235 10.1016/j.jtbi.2013.02.003

[24] Tansky M (1978) Switching effect in a prey-predator system. J Theor Biol 70: 263–271. 564991 10.1016/0022-5193(78)90376-4

[25] Pace ML, Glasser JE, Pomeroy LR (1984) A simulation analysis of continental shelf food webs. Mar Biol 8: 47–63.

[26] Ryabchenko VA, Fasham MJR, Kagan BA, Popova EE (1997) What causes short-term oscillations in ecosystem models of the ocean mixed layer? J Marine Syst 13: 33–50.

[27] Stoecker DK, Cucci TL, Hulburt EM, Yentsch CM (1986) Selective feeding by *Balanion sp*. (*Ciliata: Balanionidae*) on phytoplankton that best support its growth. J Exp Mar Biol Ecol 95: 113–130.

[28] Colton TF (1987) Extending functional response models to include a second prey type: an experimental test. Ecology 68: 900–912.

[29] Kiorboe T, Saiz E, Viitasalo M (1996) Prey switching behaviour in the planktonic copepod Acartia tonsa. Mar Ecol Prog Ser 143: 65–75.

[30] Strom SL, Loukos H (1998) Selective feeding by protozoa: model and experimental behaviors and their consequences for population stability. J Plankton Res 20: 831–846.

[31] Leising AW, Pierson JJ, Halsband-Lenk C, Horner RA, Postel J (2005) Copepod grazing during spring blooms: Does *Calanus pacificus* avoid harmful diatoms? Prog Oceanogr 67: 384–405.

[32] Nejstgaard JC, Tang KW, Steinke M, Dutz J, Koski M, et al (2007) Zooplankton grazing on *Phaeocystis*: a quantitative review and future challenges. Biogeochemistry 83: 147–172.

[33] Elliott JM (2006) Prey switching in *rhyacophila dorsalis* (trichoptera) alters with larval instar. Freshwater Biol 51: 913–924.

[34] Smout SC, Asseburg CJ, Matthiopoulos C, Fernández S, Redpath S, et al (2010) The functional response of a generalist predator. PLoS One 5(5): e10761. 20523722 10.1371/journal.pone.0010761 PMC2877704

[35] Diekmann O, Gyllenberg M, Metz JA, Nakaoka S, de Roos AM (2010) Daphnia revisited: local stability and bifurcation theory for physiologically structured population models explained by way of an example. J Math Biol 61: 277–318. 19771433 10.1007/s00285-009-0299-y

[36] Johnson PTJ, Stanton DE, Preu ER, Forshay KJ, Carpenter SR (2006) Dining on disease: how interactions between infection and environment affect predation risk. Ecology 87: 1973–1980. 16937636 10.1890/0012-9658(2006)87[1973:dodhib]2.0.co;2

[37] Fasham MJR, Ducklow HW, McKelvie SM (1990) A nitrogen-based model of plankton dynamics in the oceanic mixed layer. J Marine Res 48: 591–639.

[38] Jeschke JM, Kopp M, Tollrian R (2004) Consumer-food systems: why type I functional responses are exclusive to filter feeders. Biol Rev 79: 337–349. 15191227 10.1017/s1464793103006286

[39] Akre BG, Johnson DM (1979) Switching and sigmoid functional-response curves by damselfly naiads with alternative prey available. J Anim Ecol 48: 703–720.

[40] Dale BM, Adams LG, Bowyer RT (1994) Functional response of wolves preying on barren-ground caribou in a multiple-prey ecosystem. J Anim Ecol 63: 644–652.

[41] Elliott JM (2004) Prey switching in four species of carnivorous stoneflies. Freshwater Biol 49: 709–720.

[42] Haydon DT (1994) Pivotal assumptions determining the relationship between stability and complexity: An analytical synthesis of the stability complexity debate. Amer Nat 144: 14–29.

[43] Adjou M, Bendtsen J, Richardson K (2012) Modeling the influence from transport, mixing and grazing on phytoplankton diversity. Ecol Model 225: 19–27.

[44] Yachi S, Loreau M (1999) Biodiversity and ecosystem productivity in a fluctuating environment: the insurance hypothesis. Proc Natl Acad Sci USA 96: 1463–1468. 9990046 10.1073/pnas.96.4.1463 PMC15485

[45] Gross K, Cardinale BJ (2007) Does species richness drive community production or vice versa? Reconciling historical and contemporary paradigms in competitive communities. Amer Nat 170: 207–220. 17874372 10.1086/518950

[46] Hutchinson GE (1961) The paradox of the plankton. Amer Nat 95: 137–145.

[47] Raymont JEG (1980) Plankton and Productivity in the Oceans. Phytoplankton, vol. 1. Pergamon, Oxford.

[48] Falkowski PG, Woodhead AD (eds) (1992) Primary productivity and biogeochemical cycles in the sea. Plenum Press, New York, 550p.

[49] Irigoien X, Huisman J, Harris RP (2004) Global biodiversity patterns of marine phytoplankton and zooplankton. Nature 429: 863–867. 15215862 10.1038/nature02593

[50] Venter JC, Remington K, Heidelberg JF, Halpern AL, Rusch D., et al. (2004) Environmental genome shotgun sequencing of the Sargasso Sea. Science 304, 66–74. 10.1126/science.1093857 15001713

[51] Erwin TL (1982) Tropical forests: their richness in Coleoptera and other arthropod species. Coleopterists Bull 36: 74–75.

[52] Huston M (1985) Variation in coral growth rates with depth at Discovery Bay, Jamaica. Coral Reefs 4: 19–25.

[53] Arditi R, Ginzburg LR (1989) Coupling in predator-prey dynamics: ratio-dependence. J Theor Biol 139, 311–326.

[54] Abrams P, Ginzburg LR (2000) The nature of predation: prey dependent, ratio dependent or neither? TREE 15: 337–341. 10884706 10.1016/s0169-5347(00)01908-x

[55] Morozov AY, Arashkevich AG (2012) Towards a correct description of zooplankton feeding in models: Taking into account food-mediated unsynchronized vertical migration. J Theor Biol 262: 346–360. 10.1016/j.jtbi.2009.09.023 19782091

[56] Arditi R, Michalski J (1995) Nonlinear food web models and their reponses to increased basal productivity. In: Polis, G.A., Winemiller, K.O. (Eds.), Food webs: Integration of Patterns and Dynamics. Chapman & Hall, London, England, 122–133.

[57] Edwards AM, Brindley J (1999) Zooplankton mortality and the dynamical behavior of plankton population models. Bull Math Biol 61: 202–339. 10.1006/bulm.1998.0082 17883212

[58] Franks PJS (2009) Planktonic ecosystem models: perplexing parameterizations and a failure to fail. J Plankton Res 31: 1299–1306.

[59] Koen-Alonso M (2007) A process-oriented approach to the multi-species functional response. In: Rooney N, McCann KS, Noakes DLG, editors. From Energetics to Ecosystems: The Dynamics and Structure of Ecological Systems. Dordrecht: Springer.

[60] Real LA (1977) The kinetics of functional response. Amer Natur 111: 289–300.

[61] Teramoto E, Kawasaki K, Shigesada N (1979) Switching effect of predation on competitive prey species. J Theor Biol 79: 303–315. 522496 10.1016/0022-5193(79)90348-5

[62] Vance RR (1978) Predation and resource partitioning in one-predator–two-prey model communities. Amer Nat 112: 797–813.

[63] Evans GT, Garcón VC (*eds*) (1997) One-dimensional models of water column biogeochemistry. Joint Global Ocean Flux Study Rep. No. 23/97. JGOFS, Bergen, Norway.

[64] Koen-Alonso M, Yodzis P (2005) Multispecies modelling of some components of the marine community of northern and central Patagonia, Argentina. J Fish Aquat Sci 62: 1490–1512.

[65] Stock CA, Powell TM, Levin SA (2008) Bottom-up and top-down forcing in a simple size-structured plankton dynamics model. J Mar Syst 74: 134–152.

[66] DeMott WR (1982) Feeding selectivities and relative ingestion rates of Daphnia and Bosmina. Limnol Oceanogr 27: 518–527.

[67] Hansen B, Tande KS, Berggreen UC (1990) On the trophic fate of *Phaeocystis* pouchetii (Hariot). III. Functional responses in grazing demonstrated on juvenile stages of *Calanus finmarchicus* (Copepoda) fed diatoms and Phaeocystis. J Plankton Res12: 1173–1187.

[68] Hirst AG, Bunker AJ (2003) Growth of marine planktonic copepods: global rates and patterns in relation to chlorophyll a, temperature, and body weight. Limnol Oceanogr 48: 1988–2010.

[69] Calbet A, Landry M (2004) Phytoplankton growth, microzooplankton grazing and carbon cycling in marine systems. Limnol Oceanogr 49: 51–57.

[70] Irigoien X, Flynn KJ, Harris RP (2005) Phytoplankton blooms: a ‘loophole’ in microzooplankton grazing impact? J Plankton Res 27: 313–321.

[71] Vallina SM, Ward BA, Dutkiewicz S, Follows MJ (2012) Maximal foraging with active prey-switching: a new kill-the-winner functional response and its effect on global species richness and biogeography. Communication at ASLO meeting, 2012. Available online at http://darwinproject.mit.edu/wp-content/uploads/2012/09/asloasm2012japan_powerpoint.pdf.

[72] Naeem S, Li S (1997) Biodiversity enhances ecosystem reliability. Nature 390: 507–509.

[73] Fussmann GF, Blasius B (2005) Community response to enrichment is highly sensitive to model structure. Biol Lett 1, 9–12. 10.1098/rsbl.2004.0246 PMC1629049 17148115

[74] Morozov AY (2010) Emergence of Holling type III zooplankton functional response: bringing together field evidence and mathematical modelling. J Theor Biol 265: 45–54. 20406647 10.1016/j.jtbi.2010.04.016

[75] Cordoleani F, Nérini D, Gauduchon M, Morozov AY, Poggiale JC (2011) Structural sensitivity of biological models revisited. J Theor Biol 283: 82–91. 21641916 10.1016/j.jtbi.2011.05.021

[76] Adamson MW, Morozov A (2013) When can we trust our model predictions? Unearthing structural sensitivity in biological systems. Proc R Soc A 469: 20120500.

[77] Petrovskii SV, Morozov AY (2009) Dispersal in statistically structured population: fat tails revisited. Amer Nat 173: 278–289. 19123917 10.1086/595755

[78] Visser A, Fiksen Ø (2013) Optimal foraging in marine ecosystem models: selectivity, profitability and switching. Mar Ecol Prog Ser 473: 91–101.

