# Supplementary material for: Feeding on Multiple Sources: Towards a Universal Parameterization of the Functional Response of a Generalist Predator Allowing for Switching
Source: PLoS One. 2013 Sep 25;8(9):e74586. doi: 10.1371/journal.pone.0074586 (PMC3783441; doi:10.1371/journal.pone.0074586)
Supplement: Material S1 — Verification of the Basic Rules for the multi-prey functional responses listed in Table 1. (DOC) [file pone.0074586.s001.doc]

**Supplementary Material S1**

Here we verify whether rules (i)-(vi) formulated in section 2 are satisfied for the multi-prey functional responses listed in table 1.

**(i) Functional response from [12], [14] and [64]**

*Rule (i)* is not satisfied. Indeed, in the case where a single prey species is split into *n* groups the total intake rate will be, where $f_{0i}\left(P\right)$is a single species functional response,.

*Rule (ii)* is, obviously, not satisfied since rule (i) is not satisfied.

*Rule (iii)* is not satisfied since the difference

cannot be made as small as possible unless$P{}_{j} \approx P{}_{i}$.

*Rule (iv)*

We shall compute the stationary densities of species. The equations for the stationary densities are given by

$0=R_{i}-g_{\max}\frac{\rho_{i}P_{i}}{K\sum_{i} \rho_{i}P_{i}+\sum_{i} \rho_{i}P_{i}^{2}}Z$, (S1)

$\theta g_{\max} \frac{\sum_{i} \rho_{i}P_{i}^{2}}{K\sum_{i} \rho_{i}P_{i}+\sum_{i} \rho_{i}P_{i}^{2}}=\delta$, (S2)

where we denote $R_{i}=r_{i}\left(N\right)-\mu_{i}$. It is easy to show that *P_1_* and *P_i_* are related by the following expression

$P_{i}=\frac{\rho_{1}}{R_{1}}\frac{R_{i}}{\rho_{i}}P_{1}$ (S3)

We substitute (S3) into (S2) and obtain

$\theta g_{\max}\frac{\sum_{i} \rho_{i}\left(\frac{\rho_{1}}{R_{1}}\frac{R_{i}}{\rho_{i}}P_{1}\right)^{2}}{K\sum_{i} \rho_{i}\left(\frac{\rho_{1}}{R_{1}}\frac{R_{i}}{\rho_{i}}P_{1}\right)+\sum_{i} \rho_{i}\left(\frac{\rho_{1}}{R_{1}}\frac{R_{i}}{\rho_{i}}P_{1}\right)^{2}}=\delta$, (S4)

The solution of (S4) is given by $P_{1}=\frac{R_{1}}{\rho_{1}}\frac{K\sum_{i} R_{i}}{\sum_{i} \frac{R_{i}^{2}}{\rho_{i}}}\frac{\delta}{\left(\theta g_{\max}-\delta\right)}$,

Thus for *P_i_* we have

$P_{i}=\frac{R_{i}}{\rho_{i}}\frac{K\sum_{i} R_{i}}{\sum_{i} \frac{R_{i}^{2}}{\rho_{i}}}\frac{\delta}{\left(\theta g_{\max}-\delta\right)}$, (S5)

We can easily derive for the total biomasses of prey *P* and that of predator Z. By summing up equations (S1) we get

$\sum_{i} R_{i}=g_{\max}\frac{\sum_{i} R_{i}}{K\sum_{i} R_{i}+P_{1}\frac{\rho_{1}}{R_{1}}\sum_{i} \frac{R_{i}^{2}}{\rho_{i}}}Z$,

After some simplifications we get

$Z=\frac{\theta}{\left(\theta g_{\max}-\delta\right)}K\sum_{i} R_{i}$ (S6)

For the total biomass of prey we have

$P=\sum_{i} P_{i}=\sum_{i} \frac{R_{i}}{\rho_{i}} \frac{\sum_{i} R_{i}}{\sum_{i} \frac{R_{i}^{2}}{\rho_{i}}}\frac{K\delta^{2}}{\left(\theta g_{\max}-\delta\right)}$ (S7)

Let us now split each resource *i* into *m* groups with the close life history traits. We can do it since in reality each resource *i* combines several groups of close resources with different traits. Although *m* species obtained from resolving initial resource *i* have close life traits, they can describe morphologically different species, for each of which the given formulation of functional response should apply. The new number of species of different resources in the system will be 2*n*. It is easy to see based on (S9) that the total biomass *P* will roughly increase by 2 (*R_i_* are close to each other), which is rather a model artifact. Note that if we split each prey species in *m* cohorts, the total biomass *P* of prey will increase by the factor of *m*. This should be considered as an artifact of the model and as the intrinsic relation between the biodiversity and ecosystem productivity, rule (iv) is not satisfied.

Further we can derive the expression for the stationary density of nutrient *N*. We assume for the sake of simplicity that there is only one limiting nutrient *N* in the system. We sum up equations (7) at equilibrium (i.e. under condition *dP_i_*/*dt*=0) and obtain

$0=\sum r_{i}\left(N\right)P_{i}-\sum\mu_{i}P_{i}-\sum f_{i}Z$,

Combining with equations (6) and (8) at equilibrium gives

$$0=D_{0}\left(N_{0}-N\right)-Z\delta/\theta-\sum\mu_{i}P_{i}$$

We substitute *Z* and *P* from (S6) and (S5), respectively, and for the sake of simplicity consider that the prey mortality is small compared to predation (we can set *μ_i_ ≈*0)*.* Finally, to be able to obtain analytically tractable expressions, we consider the nutrient uptake rate by *P_i_* to be linear functions (which is a reasonable approximation for small *N*): *r_i_=a_i_N*.

$$0=D_{0}\left(N_{0}-N\right)-\frac{\theta N\delta K }{\left(\theta g_{\max}-\delta\right)\theta}\sum_{i} a_{i}$$

From which we derive the stationary concentration of nutrients *N*:

$N=\frac{D_{0}N_{0}}{D{}_{0}+\frac{\theta\delta K }{\left(\theta g_{\max}-\delta\right)\theta}\sum_{i} a_{i}}$, (S8)

In can be easily seen splitting species into groups with close life traits will result in a proportional increase in $\sum_{i} a_{i}$with an eventual drop in *N*, which is a model artefact.

On the contrary, for the total biomass of prey species, splitting into groups will result in an increase of *P*

$P=\sum_{i} P_{i}=\sum_{i} \frac{a_{i}}{\rho_{i}} \frac{\sum_{i} a_{i}}{\sum_{i} \frac{a_{i}^{2}}{\rho_{i}}}\frac{K\delta^{2}}{\left(\theta g_{\max}-\delta\right)}$ , (S9)

i.e. increasing the number of species by the factor of *m* will lead to an m-fold increase of *P* and for large number of species *n* in the system obtained by gradually resolving resources into finer groups we have

$P=\sum_{i} P_{i}\approx C\cdot n$, *C=const*. (S9’)

*Rule (v)* Consider for the sake of simplicity only 2 species (*n*=2). Differentiation of the total food intake rate gives

,

with *i≠j*, *i*, *j*=1,2. One can see that for small ratio between *P_j_* and *P_i_* the derivative will be negative, thus rule (v) is not satisfied. Considering *n*>2 one can easily arrive to the same conclusion.

*Rule (vi)* In the case of single-resource we have

,

which is clearly a Holling type II response.

**(ii) Functional response from [26]**

*Rule (i)* is not satisfied since, where $f_{0i}\left(P\right)$is a single species functional response,.

*Rule (ii)* is not satisfied since rule (i) is not satisfied.

*Rule (iii)* is not satisfied since the difference

cannot be made as small as possible unless$P{}_{j} \approx P{}_{i}$.

*Rule (iv)* The equations for the stationary densities of species are given by

, (S10)

$\theta g_{\max}\frac{\sum_{i} \rho_{i}P_{i}^{2}}{K^{2}+\sum_{i} \rho_{i}P_{i}^{2}}=\delta$, (S11)

where we denote $R_{i}=r_{i}\left(N\right)-\mu_{i}$. It is easy to show that *P_1_* and *P_i_* are related by (S3), i.e. as for the previous functional response from. We substitute (S3) into (S10) and obtain

$\theta g_{\max}\frac{\sum_{i} \rho_{i}\left(\frac{\rho_{1}}{R_{1}}\frac{R_{i}}{\rho_{i}}P_{1}\right)^{2}}{K^{2}+\sum_{i} \rho_{i}\left(\frac{\rho_{1}}{R_{1}}\frac{R_{i}}{\rho_{i}}P_{1}\right)^{2}}=\delta$, (S12)

The solution of (S12) is given by

$P_{1}=\frac{R_{1}}{\rho_{1}}\frac{K}{\left(\sum_{i} \frac{R_{i}^{2}}{\rho_{i}}\right)^{1/2}}\left(\frac{\delta}{\left(\theta g_{\max}-\delta\right)}\right)^{1/2}$, (S13)

Thus using (S3) we can derive the expressions *P_i_*

$P_{i}=\frac{R_{i}}{\rho_{i}}\frac{K}{\left(\sum_{i} \frac{R_{i}^{2}}{\rho_{i}}\right)^{1/2}}\left(\frac{\delta}{\left(\theta g_{\max}-\delta\right)}\right)^{1/2}$. (S14)

The total biomass of prey in the system can be computed as the sum of *P_i_*, this gives

$P=\sum_{i} P_{i}=\frac{K\sum_{i} \frac{R_{i}}{\rho_{i}}}{\left(\sum_{i} \frac{R_{i}^{2}}{\rho_{i}}\right)^{1/2}}\left(\frac{\delta}{\left(\theta g_{\max}-\delta\right)}\right)^{1/2}$. (S15)

As for the previous functional response, subdividing species *i* into *m* groups with close life history traits will result in the increase of *P* approximately by the factor of *m*. Rule (iv) is not satisfied.

*Rule (v)* Differentiation of the total food intake rate gives

,

It is easy to see that the derivative is always positive, thus rule (v) is satisfied.

*Rule (vi)* In the case of single-resource we have

,

which is clearly a Holling type III response.

**(iii) Functional response from [24].**

*Rule (i)* is not satisfied. In the case where a single prey species is split into *n* cohorts the total intake rate will be, where $f_{0i}\left(P\right)$is a single species functional response

*Rule (ii)* is, obviously, not satisfied since rule (i) is not satisfied.

*Rule (iii)* is not satisfied since the expression

cannot be made as small as possible unless$P{}_{j} \approx P{}_{i}$.

*Rule (iv)* is

The stationary density of phytoplankton of species *P_i_* can be obtained using the similar method as for the previous functional responses.

$P_{i}=\frac{R_{i}}{\rho_{i}}\frac{\delta\sum_{i} \frac{R_{i}^{\gamma}}{\rho_{i}^{\gamma-1}}}{g\theta\sum_{i} \frac{R_{i}^{\gamma+1}}{\rho_{i}^{\gamma}}}$. (S16)

The stationary density of the predator is given by

$Z=\frac{\sum_{i} R_{i}}{g }$, (S17)

We can easily derive for the total biomasses of prey by summing up equations (16)

$P=\sum_{i} P_{i}=\frac{\delta\sum_{i} \frac{R_{i}^{\gamma}}{\rho_{i}^{\gamma-1}}\sum_{i} \frac{R_{i}}{\rho_{i}}}{g\theta\sum_{i} \frac{R_{i}^{\gamma+1}}{\rho_{i}^{\gamma}}}$, (S18)

As for the previous functional response, subdividing species *i* into *m* groups with close life history traits will result in the increase of *P* approximately by the factor of *m*. Rule (iv) is not satisfied.

*Rule (v)* Consider for the sake of simplicity only 2 species (*n*=2). Differentiation of the total food intake rate gives (after some re-arrangement)

$\frac{\partial}{\partial P_{i}}\left(g\frac{\sum\rho_{i}P_{i}^{\gamma+1}}{\sum_{i} \rho_{i}P_{i}^{\gamma}}\right)=g\frac{\left[\left(\frac{P_{i}}{P_{j}}\right)\left(\gamma+1\right)\rho_{j}+\left(\frac{P_{i}}{P_{j}}\right)^{\gamma+1}\rho_{i}-\gamma\rho_{j}\right]}{\left(\sum_{i} \rho_{i}P_{i}^{\gamma}\right)^{2}}\frac{P_{j}^{\gamma+1}}{P_{i}}$,

with *i≠j*, *i*, *j*=1,2. One can see that for small ratio between *P_i_* and *P_j_* the derivative will be negative, thus rule (v) is not satisfied. Considering *n*>2 one can easily arrive to the same conclusion.

*Rule (vi)* In the case of single-resource we have

$f_{i}=gP_{i}$,

which is clearly a Holling type I response.

**(iv) Functional response from [62].**

*Rule (i)* is not satisfied. In the case where a single prey species is split into *n* cohorts the total intake rate will be, where $f_{0i}\left(P\right)$is a single species functional response

*Rule (ii)* is, obviously, not satisfied since rule (i) is not satisfied.

*Rule (iii)* is not satisfied since the expression

cannot be made as small as possible unless$P{}_{j} \approx P{}_{i}$.

*Rule (iv)* The stationary density of phytoplankton of species *P_i_* can be obtained using the similar method as before.

$P_{i}=\frac{R_{i}}{\rho_{i}}\frac{\delta\left(\sum_{i} R_{i}\right)^{\gamma}}{g\theta\sum_{i} \frac{R_{i}^{\gamma+1}}{\rho_{i}^{\gamma}}}$, (S19)

We can easily derive for the total biomasses of prey by summing up equations (S18).

$P=\sum_{i} P_{i}=\delta\frac{\sum_{i} \frac{R_{i}}{\rho_{i}} \left(\sum_{i} R_{i}\right)^{\gamma}}{g\theta\sum_{i} \frac{R_{i}^{\gamma+1}}{\rho_{i}^{\gamma}}}$, (S20)

As for the previous functional response, subdividing species *i* into *m* groups with close life history traits will result in the increase of *P* approximately by the factor of *m*. Rule (iv) is not satisfied.

*Rule (v)* Consider for the sake of simplicity only 2 species (*n*=2). Differentiation of the total food intake rate gives (after some re-arrangement)

$\frac{\partial}{\partial P_{i}}\left(g\frac{\sum\rho_{i}P_{i}^{\gamma+1}}{\left(\sum_{i} \rho_{i}P_{i}\right)^{\gamma}}\right)=g\frac{\left[\rho_{j}\left(\gamma+1\right)\frac{P_{i}}{P_{j}}+\rho_{j}^{2}-\gamma\rho_{i}\rho_{j}\frac{P_{i}^{\gamma+1}}{P_{j}^{\gamma+1}}\right]}{\left(\sum_{i} \rho_{i}P_{i}\right)^{\gamma+1}}P_{2}^{\gamma+1}$,

One can see that for large ratio between *P_i_* and *P_j_* the derivative will be negative, thus rule (v) is not satisfied. Considering *n*>2 one can easily arrive to the same conclusion.

*Rule (vi)* In the case of single-resource we have

$f_{i}=gP_{i}$,

which is clearly a Holling type I response.

**(v) Functional response from [65]**

$f_{i}=g_{\max}\frac{\varphi_{i}P_{i}}{K+\sum_{i} \varphi_{i}P_{i}}$, with $\varphi_{i}=\left(\frac{\left(\rho_{i}P_{i}\right)^{m}}{\sum_{i} \left(\rho_{i}P_{i}\right)^{m}}\right)^{\gamma}$

This functional response is actually a generalization of functional responses (i), (iii) and (iv).

In a similar way as for responses (i), (iii) and (iv) one can prove that none of the basic rules (i)-(v) is valid in the case *m*≠0.

**(vi) Functional response of [63]**

*Rule (i)* is not satisfied. Indeed, in the case where a single prey species is split into *n* groups the total intake rate will be , where $f_{0i}\left(P\right)$is a single species response,, *δ* >0.

*Rule (ii)* is, obviously, not satisfied since rule (i) is not satisfied.

*Rule (iii)* is not satisfied since the difference

cannot be made as small as possible unless$P{}_{j} \approx P{}_{i}$ or all *δ_i_* =0.

*Rule (iv)*

The stationary density of phytoplankton of species *P_i_* can be obtained using the similar method as before.

$P_{i}=\frac{R_{i}N-R{}_{1}}{R_{1}\delta_{i}}$, (S21)

We can easily derive for the total biomasses of prey by summing up equations (S21).

$P=\sum_{i} P_{i}=\frac{1}{R_{1}}\sum\frac{R_{i}N-R_{1}}{\delta_{i}}$, (S22)

As for the previous functional response, subdividing species *i* into *m* groups with close life history traits will result in the increase of *P* approximately by the factor of *m*. Rule (iv) is not satisfied.

*Rule (v)* Differentiation of the total food intake rate gives (after some re-arrangement)

$$\frac{\partial}{\partial P_{i}}\left(g_{\max}\frac{\sum P_{i}\left(1+\delta_{i} P_{i}\right)}{K+\sum_{i} P_{i}\left(1+\delta_{i} P_{i}\right)}\right)=g_{\max}\frac{K}{\left(K+\sum_{i} P_{i}\left(1+\delta_{i} P_{i}\right)\right)^{2}}\left(1+2P_{i}\right)>0$$

Thus, rule (v) is satisfied.

*Rule (vi)* In the case of single-resource we have

$f_{i}=g_{\max}\frac{P_{i}\left(1+\delta P_{i}\right)}{K+P_{i}\left(1+\delta P_{i}\right)}$,

To define a Holling type I of *f_i_* we shall analyse the behaviour of the clearance rate, i.e. the ratio *f_i_*/*P_i_*. A straightforward computation shows that *f_i_*/*P_i_* always decrease in the case *Kδ*<1. Thus, in the case *Kδ*<1 the single functional response is of Holling type II, in the case *Kδ*>1 the functional response is of Holling type III.

**(vii) Functional response given by equation (10) (this paper, see also [71])**

*Rule (i)* is satisfied. In the case where a single prey species is split into *n* cohorts the total intake rate will be, where $f_{0i}\left(P\right)$is a single species response

*Rule (ii)* is not satisfied. Indeed, consider two different resources *P_1_* and *P_2_* and split *P_1_* into *m* groups. Before splitting the intake rate of resource *P_1_* is given by

$$f_{1}\left(P_{1},P_{2}\right)=\frac{\rho_{1}P_{1}^{\gamma}}{\sum_{i} \rho_{i}P_{i}^{\gamma}}F\left(\sum_{i} \rho_{i}P_{i}\right)$$

After splitting the intake rate of resource 1 (*P_1_* = *P_11_*+ *P_12_*+…+ *P_1m_*) is given by

$\sum_{j=1}^{m} f_{1 j}\left(P_{11},P_{12},\ldots,P_{1m},P_{2}\right)=\sum_{j} \rho_{1}P_{1j}^{\gamma}\frac{1}{\sum_{i,j} \rho_{i}P_{ij}^{\gamma}}F\left(\sum_{i} \rho_{i}P_{i}\right)\neq\frac{\rho_{1}P_{1}^{\gamma}}{\sum_{i} \rho_{i}P_{i}^{\gamma}}F\left(\sum_{i} \rho_{i}P_{i}\right)=f_{1}\left(P_{1},P_{2}\right)$*Rule (iii)* is not satisfied since the expression

cannot be made as small as possible unless$P{}_{j} \approx P{}_{i}$and *γ*=1. In the latter case there is no active switching of predator and the consumption is proportional-based.

*Rule (iv)*

For the stationary densities of prey *i* we have

$P_{i}=\frac{\left(\frac{R_{i}\rho_{1}}{\rho_{i}R_{1}}\right)^{1/(1-\gamma)}}{\sum_{i} \rho_{i}\left(\frac{R_{i}}{\rho_{i}}\right)^{1/(1-\gamma)}}F^{-1}\left(\frac{\delta}{\theta}\right)$, (S23)

where we denote $R_{i}=r_{i}\left(\bar{N}\right)()-\mu_{i}$. The total biomass *P* of prey is given by

$P=\sum_{i} P_{i}=\frac{\sum_{i} \left(\frac{R_{i}\rho_{1}}{\rho_{i}R_{1}}\right)^{1/(1-\gamma)}}{\sum_{i} \rho_{i}\left(\frac{R_{i}}{\rho_{i}}\right)^{1/(1-\gamma)}}F^{-1}\left(\frac{\delta}{\theta}\right)$ (S24)

It is easy to show that splitting each species into the same number *m* of subpopulations with the close life traits will not affect the value of *P*: for the densities *P_ij_* of subpopulations we shall have *P_ij_* = *P_i_*/*m* as it should be the case. However, a major problem arises in the case where we split *P_i_* into different number of subpopulations *m_i_*. Consider for the sake of simplicity the situation where there are only two prey species. We split resource1 and 2 into *m_1_* and *m_2_* subparts, respectively. Obviously, due to the symmetry for the stationary densities of subpopulations we should have *P_ij_* = *P_i_*/*m_i_*. Surprisingly enough, this is not the case in the given model. Indeed, for *P_ij_* we use (S23) and obtain (assuming *m_2_>m_1_*)

$P_{1j}=\frac{1}{m_{1}\sum_{i} \rho_{i}\left(\frac{R_{i}}{\rho_{i}}\right)^{1/(1-\gamma)}+\left(m_{2}-m_{1}\right)\rho_{2}\left(\frac{R_{2}}{\rho_{2}}\right)^{1/(1-\gamma)}}F^{-1}\left(\frac{\delta}{\theta}\right)<\frac{P_{1}}{m_{1}}$, (S25)

$P_{2j}=\frac{\left(\frac{R_{2}\rho_{1}}{\rho_{2}R_{1}}\right)^{1/(1-\gamma)}}{m_{2}\sum_{i} \rho_{i}\left(\frac{R_{i}}{\rho_{i}}\right)^{1/(1-\gamma)}-\left(m_{2}-m_{1}\right)\rho_{1}\left(\frac{R_{1}}{\rho_{1}}\right)^{1/(1-\gamma)}}F^{-1}\left(\frac{\delta}{\theta}\right)>\frac{P_{2}}{m_{2}}$, (S26)

One can easily see that summing back the subpopulations over the cohorts does not result in the same stationary densities *P_1_* and *P_2_*. Moreover, by considering a large number of subpopulations of the second prey species (*m_2_>>m_1_*), we will arrive to the paradoxical situation: species 1 should simply extinct (see (S25)). This is, definitely, an artifact of the parameterization. Note such a discrepancy can affect the primarily production of the system. Indeed, if we intentionally consider a very large number of subspecies with small growth rates *R_i_* and regroup the other prey species with higher *R_i_*, the relative abundance of species with small growth rates will be much higher due to the way we performed our grouping of species. Thus, the primary production (which is the sum of all *P_i_ R_i_* ) will be simply the result of our choice of grouping and portioning of species. Thus *rule (iv)* is not satisfied.

*Rule (v)* Differentiation of the total food intake rate gives (after some re-arrangement)

$$\frac{\partial}{\partial P_{i}}\left(F\left(\sum\rho_{i}P_{i}\right)\right)>0$$

Thus, rule (v) is satisfied since we consider *F* to be an increasing function.

*Rule (vi)* In the case of single-resource the Holling type is entirely determined by the choice of *F*.

**(viii) Functional response from [23]**

Note that we slightly change the initial mathematical notation from van Leeuwen et al., 2013 replace *T* with 1/*K*, where *K* the half saturation constant and *T* is the handling time (which we assume to be the same for all resources the sake of simplicity).

*Rule (i)* is satisfied. In the case where a single prey species is split into *n* groups the total intake rate will be, where $f_{0i}\left(P\right)$is a single species response and we can set *ε_ii_*=1, *ρ*=1.

*Rule (ii)* is satisfied. Consider splitting resource *i* into *m* groups in the case other resources are present.

The overall intake rate of resource *i* (*P_i_* = *P_i1_*+ *P_i2_*+…+ *P_1m_*) is given by

$\sum_{j=1}^{m} f_{i j}\left(...P_{i1},P_{i2},\ldots,P_{1m},...\right)=g_{\max}\frac{P_{i}\rho_{i}\sum_{j} \rho_{j}\varepsilon_{ij}P_{j}}{\sum_{i} \rho_{i}P_{i}\left(K+\sum_{j} \rho_{j}\varepsilon_{ij}P_{j}\right)}=f_{i}\left(...P_{i}...\right)$,

since all coefficients *ε_ij_* and *ρ_i_* are the same for each *P_i1_*,  *P_i2_*, …, *P_1m_*.

*Rule (iii)* is satisfied since the expression

since the set of coefficients *ε_ij_* will be close to each other.

*Rule (iv)* is satisfied. The proof can be done in a similar way as for the universal parameterization (ix), see below.

*Rule (v)* Consider for the sake of simplicity only 2 species (*n*=2). Differentiation of the total food intake rate gives (after some re-arrangement)

$$\frac{\partial}{\partial P_{i}}\left(g_{\max}\frac{P_{i}\rho_{i}\sum_{j} \rho_{j}\varepsilon_{ij}P_{j}}{\sum_{i} \rho_{i}P_{i}\left(K+\sum_{j} \rho_{j}\varepsilon_{ij}P_{j}\right)}\right)=g_{\max}\frac{\left(2\rho_{i}^{2}\left(2\varepsilon_{ij}-\varepsilon_{ii}\right)\left(\frac{P_{i}}{P_{j}}\right)^{2}+2\rho_{1}\rho_{2}\varepsilon_{ii}\frac{P_{i}}{P_{j}}+\rho_{j}^{2} \varepsilon_{ii}\right)}{\left[\sum_{i} \rho_{i}P_{i}\left(K+\sum_{j} \rho_{j}\varepsilon_{ij}P_{j}\right)\right]^{2}}\rho_{j}KP_{j}^{2}$$

with *i≠j*, *i*, *j*=1,2, we consider that $\varepsilon_{ij}=\varepsilon_{ji}$ One can see that for large ratio between *P_i_* and *P_j_* the derivative will be negative, thus rule (v) is not satisfied in the case $2\varepsilon_{ij}<\varepsilon_{ii}$. On the contrary, the opposite constrain $2\varepsilon_{ij}>\varepsilon_{ii}$seriously undermines active switching effect resulting in proportion-based consumption (see section 3 of the main text for detail).

*Rule (vi)* In the case of single-resource we have

$f_{i}=g_{\max}\frac{P_{i}\varepsilon_{ii}\rho_{i}}{K+P_{i}\varepsilon_{ii}\rho_{i}}$,

which is clearly a Holling type II response.

**(ix) Universal parameterization of functional response (this paper, equations (12) and (15)).**

*Rules (i) - (iii)* are satisfied since for resources with close life traits parameterization (15) is equivalent to a proportion-based consumption (see section 4 for detail) and for proportion-based parameterizations rules (i)-(iii) are always satisfied (see functional response (x)).

*Rule (iv)*. Analytical investigation of model (6)-(8) with the given functional response is complicated issue since the mathematical formulation depends on a large number of parameters, in particular, the parameter *σ*, which quantifies the width of the kernel. For instance, for large *σ* the functional response becomes equivalent to the proportion-based response (13) for which the co-existence of several prey species is impossible. On the other hand, for small *σ* (15) is equivalent to (10) (see section 4) for which the coexistence of multiple prey species was demonstrated above and the expressions for the stationary densities were found (see (S23)). A comprehensive study of the existence of stationary states for intermediate values of *σ* should be found elsewhere. Our preliminary study based on numerical analysis has revealed the existence of coexistence state (not shown result).

Here we can easily prove that for the universal functional response (15) the splitting populations in groups will not affect the actual stationary densities and, thus the total biomass in the ecosystem as well as the ecosystem’s productivity.

We consider the equation for the prey species *i* which reads as follows

, (S27)

We split *P_i_* into *m_i_* cohorts. For each cohort we shall have similar equation.

, (S28)

Obviously, summing up equations (S28) will result in equation (S27) for *P_i_* due to rule (ii) which is satisfied for the universal parameterization (15). The same holds true for summing up the case of slightly different subpopulations.

*Rule (v)* Differentiation of the total food intake rate gives (after some re-arrangement)

$$\frac{\partial}{\partial P_{i}}\left(F\left(\sum\rho_{i}P_{i}\right)\right)>0$$

Thus, rule (v) is satisfied since we consider *F* to be an increasing function.

*Rule (vi)* In the case of single-resource the Holling type is entirely determined by the choice of *F*.

**(x) Proportion-based response from [11].**

*Rule (i)* is satisfied. In the case where a single prey species is split into *n* cohorts the total intake rate will be, where $f_{0i}\left(P\right)$is a single species functional response. Here $F(P)=g_{\max}\frac{P}{K+P}$

*Rule (ii)* is satisfied as well since splitting *P_i_* in to m cohorts and summing the intake rates gives the same result:

$\sum_{j=1}^{m} f_{i j}\left(P_{1},\ldots P_{i1},\ldots,P_{1m},\ldots P_{n}\right)=\sum_{j} \rho_{i}P_{ij}\frac{1}{\sum_{i,j} \rho_{i}P_{ij}}F\left(\sum_{i} \rho_{i}P_{i}\right)=\frac{\rho_{i}P_{i}}{\sum_{i} \rho_{i}P_{i}}F\left(\sum_{i} \rho_{i}P_{i}\right)=f_{i}\left(\vec{P}\right)()$*Rule (iii)* is satisfied since the expression

for close species with$\rho_{i}\approx\rho_{j}$.

*Rule (iv)* is not applicable for the given since there can be no coexistence stationary state for *n*>2 prey species in (6)-(8). This is an example of the competitive exclusion principal.

*Rule (v)* is satisfied: differentiation of the total food intake rate gives

$$\frac{\partial}{\partial P_{i}}\left(F\left(\sum\rho_{i}P_{i}\right)\right)>0$$

*Rule (vi)* In the case of single-resource the Holling type is entirely determined by the choice of *F*.
